# Cold hardiness increases with age in juvenile *Rhododendron* populations

**DOI:** 10.3389/fpls.2014.00542

**Published:** 2014-10-16

**Authors:** Chon-Chong Lim, Stephen L. Krebs, Rajeev Arora

**Affiliations:** ^1^Department of Horticulture, Iowa State University, Ames, IA, USA; ^2^David G. Leach Research Station of The Holden Arboretum, Madison, OH, USA

**Keywords:** cold acclimation, chronological age, dehydrin, freezing tolerance, physiological age, *R. catawbiense*, *R. fortunei*, *R. maximum*

## Abstract

Winter survival in woody plants is controlled by environmental and genetic factors that affect the plant’s ability to cold acclimate. Because woody perennials are long-lived and often have a prolonged juvenile (pre-flowering) phase, it is conceivable that both chronological and physiological age factors influence adaptive traits such as stress tolerance. This study investigated annual cold hardiness (CH) changes in several hybrid *Rhododendron* populations based on *T*_max_, an estimate of the maximum rate of freezing injury (ion leakage) in cold-acclimated leaves from juvenile progeny. Data from F_2_ and backcross populations derived from *R. catawbiense* and *R. fortunei* parents indicated significant annual increases in *T*_max_ ranging from 3.7 to 6.4°C as the seedlings aged from 3 to 5 years old. A similar yearly increase (6.7°C) was observed in comparisons of 1- and 2-year-old F_1_ progenies from a *R. catawbiense* × *R. dichroanthum* cross. In contrast, CH of the mature parent plants (>10 years old) did not change significantly over the same evaluation period. In leaf samples from a natural population of *R. maximum*, CH evaluations over 2 years resulted in an average *T*_max_ value for juvenile 2- to 3-year-old plants that was 9.2°C lower than the average for mature (~30 years old) plants. A reduction in CH was also observed in three hybrid rhododendron cultivars clonally propagated by rooted cuttings (ramets)—*T*_max_ of 4-year-old ramets was significantly lower than the *T*_max_ estimates for the 30- to 40-year-old source plants (ortets). In both the wild *R. maximum* population and the hybrid cultivar group, higher accumulation of a cold-acclimation responsive 25 kDa leaf dehydrin was associated with older plants and higher CH. The feasibility of identifying hardy phenotypes at juvenile period and research implications of age-dependent changes in CH are discussed.

## INTRODUCTION

Woody perennials typically go through a juvenile period of growth in which they remain vegetative and do not respond to flowering stimuli. This phase of development may last a few weeks in some plants to up to decades in certain shrub and tree species (Kramer and Kozlowski, 1979; Hartmann et al., 1997). Juvenility and the associated phase-change to maturity have strong influences on morphogenesis and tissue differentiation resulting in a number of morphological, physiological, and biochemical alterations in plants (Robinson and Wareing, 1969; Hackett, 1985; Zimmerman et al., 1985; Hackett et al., 1990; Poethig, 1990; Hand et al., 1996). Little is known, however, about the effect of age on abiotic stress tolerance, such as freeze or cold hardiness (CH) of plant tissues.

Improvement in the CH of woody perennials for their suitability in northern climates has largely been attempted via classical plant breeding. This includes recurrent phenotypic selection of juvenile progenies either based on their winter survival in the field or via laboratory-based CH tests which may also include evaluation of cold acclimation (CA)—the timing, rate, and degree of CH increases during fall under inductive short-days and low temperatures. In woody plant breeding programs with long generation times, there is large benefit in time and efficiency if selection can be practiced during the early stages of growth and development for traits that are required at maturity. However, the critical question of how well the expression of adaptive traits during the juvenile stage predicts mature plant phenotypes has received little attention. Anecdotal evidence indicates that field-grown juvenile seedlings of several landscape woody perennials are typically not as cold-hardy as their physiologically mature counterparts (Pellet, 1998; McNamara and Pellet, 2000), however, published literature on the relationship between juvenility, aging, and CH is scarce. Brown and Bixby (1976) observed that 1-month-old, cold-acclimated black locust (*Robinia pseudoacacia*) seedlings were killed at 0°C, whereas similarly treated 3-month-old seedlings could survive –20°C. McNamara and Pellet (2000) noted that stem tissues of 10- to 34-month-old *Phellodendron sachalinense* seedlings (half-sib populations) were hardy to ~–5°C in early October compared to –14°C for the mature female parent growing under similar conditions. Both these studies examined age-related CH only in a given year and no systematic study has been undertaken to investigate such changes over multiple years.

Indications of age-dependent CH phenotypes have been observed in prior research in genus *Rhododendron*, where the duration of juvenility is approximately 5 years from germination. In one segregating F_2_ population derived from self-pollination, the mean *T*_max_ for 2- to 3-year-old progenies was ~12°C lower than the mature parent plant grown in similar conditions (Lim et al., 1998b). For a quantitative trait such as CH, this parent-offspring discrepancy in phenotypes is difficult to account for genetically because the expectation for this population is for no change in mean value. Alternatively, it may be explainable by the dependency of CH on age (chronological and/or physiological).

Knowledge about age-dependent phenotypic expression would be useful to breeders of woody plants who would like to select individuals as early as possible for adaptive traits without having to grow the entire population to maturity. For example, mapping of quantitative trait loci and development of marker assisted selection tools would be most efficient if applied to screenings of young, often juvenile progeny. Success of this application, however, would require that linkage relationships between traits and markers remain consistent as phenotypes change with age. Age-dependent expression of traits may also impact the horticulture industry, which relies heavily on the distribution of relatively young (1- to 2-year-old), vegetatively propagated clones of woody ornamentals, shade or fruit trees, either through rooting stem cuttings or tissue culture. The adaptive phenotypes of these young propagules (ramets) may differ from the older source plants (ortets) from which they are derived.

The present study explores this concept by investigating annual changes in CH of juvenile seedlings and mature plants over two to three consecutive years in naturally occurring *Rhododendron* populations as well as breeding populations derived from interspecific hybridization.

## MATERIALS AND METHODS

### TEST PLANTS

This study involved naturally cold-acclimated plants from four different *Rhododendron* populations comprising evergreen, non-scaly leaved species within subgenus *Hymenanthes* (Goetsch et al., 2005). Three of the populations (Table 1) were derived from interspecific cross-pollinations of the cold-hardy native species *R. catawbiense* (using a selected form *R. catawbiense* “Catalgla”) with Asian relatives that are less hardy, such as *R. fortunei* and *R. dichroanthum*, for the purpose of producing progeny segregating for CH. Pedigrees for the F_1_, F_2_, and backcross (BC) populations and the number of progeny are given in Table 1. All the plants belonging to the hybrid populations and their parents were field grown at The Holden Arboretum’s David G. Leach Research Station, Madison, OH, USA. The parent plants were mature 30- to 40-year-old individuals, with the exception of *R. dichroanthum* “Sonata” which was estimated to be ~10 years old. Additional comparisons between seedling and mature plants were made using another native species, *R. maximum*, from a population of seedlings naturally growing near the Cooper’s Rock State Forest, West Virginia, USA.

**Table 1:** **Cold hardiness estimates (*T*_max_) of rhododendron cultivars and species used as parents in interspecific hybrid populations and of the resulting seedling populations at various juvenile ages.** Additional descriptions are given in Section “Materials and Methods.”

Population^*^	Parent *T*_max_ (°C)^**^		*N*	Mean progeny *T*_max_ ± SE (°C)^***^				
	♀	♂		1-year-old	2-year-old	3-year-old	4-year-old	5-year-old
F_1_	–52.4	–16.6	12	–16.1 ± 0.6^a^	–22.8 ± 2.1^b^	–	–	–
F_2_	–43.0	50	–	–	–27.5 ± 0.8^a^	–33.9 ± 0.9^b^	–
BC	–43.0	–32.0	20	–	–	–19.0 ± 1.5^a^	–22.7 ± 0.6^b^	–28.3 ± 0.9^c^

* F_1_ = R. catewbiense “Catalgla” × R. dichroanthum hybrid “Sonata.” F_2_ = R. “Ceylon” self-pollinated (R. “Ceylon” = R. catawbiense “Catalgla” × R. fortunei). BC = R. “Ceylon” × R. fortunei combined with an equal number of seedlings from the reciprocal cross.

** T_max_ values reported in Lim et al. (1998b, 1999). The value for R. dichroanthum is the average of 1997 and 1998 T_max_ determinations, which were not significantly different (data not shown).

*** For within-population comparisons (rows), mean values followed by different letter in superscripts are significantly different (P < 0.05, t-test).

### COLD HARDINESS DETERMINATION

For the hybrid populations (Table 1), uniform-sized, current-year leaves of the same progeny population were screened for two to three consecutive years. In the study of wild *R. maximum* (Table 2), different juvenile and mature individuals (but with approximately similar age difference) were scored over 2 years. Leaves were collected in December each year of the study, at which time they are cold-acclimated, and kept on ice until processed, usually within 24 h. Punched leaf disks (1 cm diameter, 24 disks per plant) were slowly cooled, following ice-nucleation at –1°C, to eight subfreezing treatment temperatures (three disks per temperature). A temperature treatment range was achieved by placing test tubes containing single disks in a temperature-controlled glycol bath (tissues cooled to –24°C) followed by transfer to a deep freezer (–24 to –52°C) as detailed in Lim et al. (1998b). Cooling rates were –1°C/30 min up to –10°C followed by ~–3.5°C/30 min thereafter. Freeze-thaw injury was estimated by measuring electrolyte leakage from frozen and overnight thawed tissues as described in Lim et al. (1998a). Ion leakage data transformations, Gompertz functions fitting, and statistical analyses were performed as described by Lim et al. (1998a) to obtain values for *T*_max_, the temperature at which the maximum rate of freezing injury occurred and the parameter used to estimate CH. In related studies of *Rhododendron* by our group, *T*_max_ has been shown to correlate highly with LT_50_, another widely used CH parameter based on ion-leakage and/or visual observations of freeze-thaw injury in rhododendron leaves (Lim et al., 1998a). Estimated CH in this study is synonymous with leaf freezing tolerance (LFT) in earlier reports.

**Table 2:** **Leaf freezing-tolerance (*T*_max_) differences between juvenile and mature plants of natural Rhododendron maximum populations.**

*R. maximum* (natural population)	*N^z^*	Mean *T_max_* (°C) ± SE^y^
Juvenile seedlings	12	–37.6 ± 1.7^b^
Mature plants	6	–46.8 ± 1.6^a^

z N, number of plants. Three replicate disks were measured at each treatment temperature.

y Estimated by using Gompertz function fitted to % adjusted injury data, mean, and SE estimated by replicates (24 leaf disks) using the Jackknife method (Lim et al., 1998a). Mean separation in column by multiple t-test, significant at P < 0.05. Mean values followed by a different letter in superscript are significantly different (P < 0.05, t-test).

## RESULTS

### F_1_ POPULATION (TABLE 1)

With the exception of *R. dichroanthum*, the *T*_max_ values of the parents used in these crosses have previously been reported (Lim et al., 1998b, 1999). The *T*_max_ of the parent *R. dichroanthum* “Sonata” did not vary significantly year to year (–17.5 and –15.7°C, respectively) with an average *T*_max_ of –16.6°C. The average *T*_max_ for 1-year-old F_1_ progeny (–16.1°C) was about the same as the cold-sensitive *R. dichroanthum* parent and 36.3°C less hardy than the *R. catawbiense* parent. The mean *T*_max_ in this population increased significantly (6.7°C) after a second year of growth.

### F_2_ POPULATION (TABLE 1)

**FIGURE 1:**
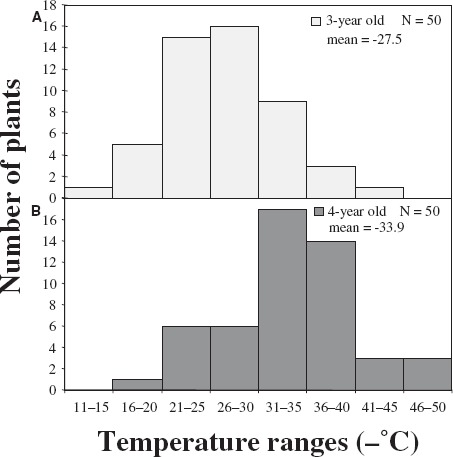
**Cold hardiness (*T*_max_) progeny distributions over two consecutive years of an F_2_ population derived from self-pollination of *R. “Ceylon,” an F_1_ selection of R. catawbiense × R. fortunei (A)* December 1996; (B) December 1997.** *N,* number of plants.

Comparison of 3- versus 4-year-old seedlings in this population revealed a significant 6.4°C increase in mean *T*_max_ in older plants, from –27.5°C at age 3 to –33.9°C at age 4. Frequency distributions of this population (Figure 1) show a normally distributed curve centered around mean *T*_max_ values that shift toward greater CH after an additional year, although still below the mature parental value of –43.0°C The distribution of F_2_ phenotypes is consistent with a polygenic control of CH that has been reported for *Rhododendron* and other taxa (Guy, 1990; Lim et al., 1998b; Pellet, 1998). The magnitude of the CH increase varied randomly among F_2_ individuals and was not correlated with an individual’s position in the CH distribution (data not shown).

### BACKCROSS POPULATION (TABLE 1)

**FIGURE 2:**
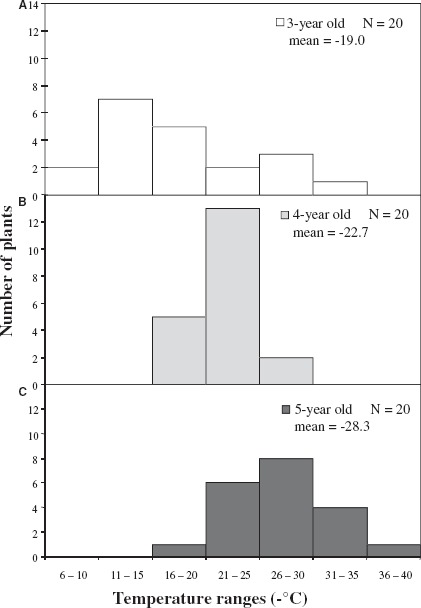
**Cold hardiness (*T*_max_) distribution of a BC population over three consecutive years (A-C).**

*T*_max_ from 10 progeny in two reciprocal BC populations— *R. fortunei* × *R.* “Ceylon” and *R.* “Ceylon” × *R. fortunei*— were annually determined between 1 and 3 years of age. Because the difference between the variances in mean *T*_max_ was nonsignificant in the reciprocal crosses (*F*-test not shown), the *T*_max_ data were pooled as a single BC population (*n* = 20). Similar progeny scores from the reciprocal crosses suggest that there is no maternal inheritance determining CH. Average *T*_max_ values in the BC population were 8.5 and 11.2°C less hardy than F_2_ populations of the same age—3 and 4 years old, respectively. Although both groups are derived from *R. catawbiense* and *R. fortunei*, the BC population has 25% more contribution from *R. fortunei*, the less cold-hardy parent, than the F_2_. Like the F_2_ population, progeny at 3, 4, and 5 years of age varied continuously for *T*_max_ (Figure 2), becoming progressively more cold-hardy. The increases in CH were significant—3.7°C (from 3- to 4-year-old) and 5.6°C (from 4- to 5-year-old). By the fifth year, some of the BC seedlings had set flower buds, an indication of physiological maturity (phase change). However, average CH in the 5-year-old progeny (–28.2°C) was still lower than mature CH of the more cold-sensitive parent, *R.* “Ceylon” (–32.0°C).

### NATURAL POPULATION (TABLE 2)

The *T*_max_ of juvenile seedlings of the *R. maximum* population was –37.6°C, significantly less hardy than older, mature plants in close proximity from the same population, which averaged –48.6°C. These results are based on the data from two consecutive years pooled together.

### CH RANK CORRELATIONS (FIGURES 3 and 4)

**FIGURE 3:**
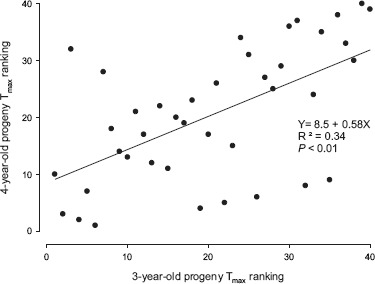
**Regression of *T*_max_ rank in 4-year-old F_2_ seedlings (*n* = 50) on a similar ranking for the same group of 3-year-old progenies.** Lower numbers have higher cold hardiness.

**FIGURE 4:**
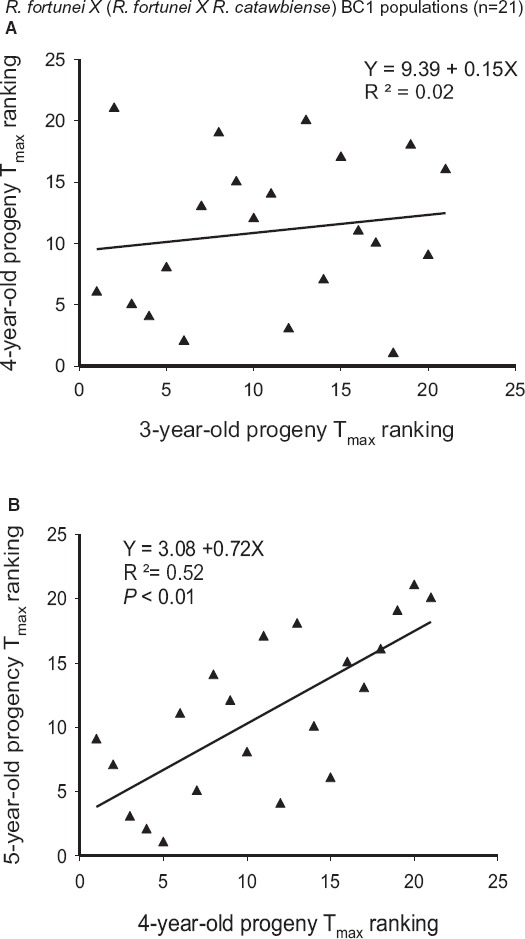
**Regression of *T*_max_ rankings over 3 years in the BC population [*R. fortunei* × (*R. fortunei* × *R. catawbiense*); *n* = 21]. (A)** Regression of 4- and 3-year-old progeny rankings. **(B)** Regression of 5- and 4-year-old progeny rankings. Lower numbers have higher cold hardiness.

In three comparisons, progeny from the same population were numerically ranked based on their *T*_max_ values, and regression plots were generated using ranks from consecutive years. In the F_2_ population (Figure 3), there was a significant correspondence in *T*_max_ values at age 3 and age 4 (*R*^2^ = 0.34, *P* < 0.01). Similar analysis of the BC population at 3 and 4 years old resulted in no correlation (Figure 4A), but in subsequent years (ages 4 and 5, Figure 4B) the CH rankings were again significantly correlated (*R*^2^ = 0.52, *P* < 0.01).

## DISCUSSION

### COLD HARDINESS INCREASES WITH AGE DURING JUVENILE PHASE

Our study used *Rhododendron* populations segregating for CH to examine the relationship between juvenile and mature CH phenotypes. Because CH is a quantitative trait in *Rhododendron* and many other taxa (Guy, 1990; Lim et al., 1998b; Pellet, 1998), average progeny phenotypes are expected to be intermediate to the parental values for CH. Our data indicate that the average *T*_max_ of 1- to 5-year-old juvenile populations is often lower than the corresponding value of the least hardy parent used in the cross, a result that corresponds with earlier findings (Lim et al., 1998b, 1999). In contrast, *T*_max_ values for physiologically mature (>10- year-old) parent plants did not change over the same evaluation period. This suggests that maximal CH in young *Rhododendron* offspring may not be fully achieved until after flowering, which is approximately 5 years from germination for seedlings grown under natural conditions.

The CH data from the F_1_, F_2_, and BC progenies indicate that juvenile *Rhododendron* seedlings gain from 3.7 to 6.7°C/year in CH, an average of 5.6°C/year. Since in this study CH was evaluated in winter (December) using field-grown plants, the increase in *T*_max_ most likely reflects a gain in the CA ability, i.e., CH of CA tissues (winter) minus the CH at non-acclimated (NA; summer) state. An earlier study of NA- and CA-*T*_max_ using a similar set of F_2_ and BC *Rhododendron* populations demonstrated that, of these two components, it is the variation in CA ability rather than NACH level that accounts for most of the segregation in *T*_max_ values among progeny, and the two components are independent of each other (Lim et al., 1998b).

Cold acclimation in temperate zone woody perennials occurs in two stages: an initial, somewhat smaller increase in the hardiness in response to the inductive short-days in early autumn, followed by a much pronounced gain induced by cold/freezing temperatures in late autumn/winter (Arora et al., 1992). Response to either environmental cue may vary with age. A study of *Phellodendron* (corktree) seedlings and mature trees in Minnesota determined that CH in early October (October 9) did not vary among 10- and 36-month-old juvenile seedlings, whereas the older progeny were three times more hardy by October 26 (McNamara and Pellet, 2000). Assuming that the seedlings were experiencing more frosts later in the fall, these data suggest that plant age in *Phellodendron* has limited effect on the photoperiod response during CA, but a large effect on how plants acclimate in response to cold temperature cues. However, the absence of presumed frosts would suggest that older seedlings (by only 2 years) were perhaps more responsive to short days for CA. Further studies with a wider array of woody plant taxa are needed to determine the extent to which age affects physiological response to the environment during CA.

### CORRESPONDENCE OF JUVENILE-MATURE PHENOTYPES

In woody plant breeding programs, genetic gains over time are slowed if the selection cycle is lengthy. In *Rhododendron*, the selection cycle is a minimum of 5 years, the time required for most seed-grown progeny to reach flowering, a key ornamental trait. By contrast, the selection cycle in conifer tree breeding is 20-25 years, the time required to reach a harvestable size (yield). Because of the economic significance of timber crops, it is not surprising that the most extensive data on age-age trait correlations derives from long-term (25+ years) experimental forestry plots. Studies of key biometric traits associated with yield, particularly tree height, have demonstrated strong genetic and phenotypic correlations between young (3- to 10-year-old) and mature (20- to 25-year-old) trees (Lambeth, 1980; McKeand, 1988). These studies also determined that trait correlation in very young plants aged 1-3 years was much weaker, possibly due to juvenility or the fact that young transplanted seedlings were still adapting to the experimental nursery sites.

The age-age CH correlations for *Rhododendron* were determined over a few years of juvenile growth and do not include progeny CH at maturity. Taken together, the results of progeny rank correlations in the F_2_ and BC populations suggest that there is moderate age-age correlation in CH rankings, and that CH selections made earlier would correspond reasonably well to selections made a year later. It remains to be determined how well these juvenile CH estimates predict winter performance at maturity.

Because the oldest population in this study (5-year-old BC seedlings) had an average *T*_max_ ~4°C below the less hardy parent, it is probable that *Rhododendron* seedlings do not attain a maximal or full CA ability until after they reach reproductive maturity (~5 years). In contrast, McNamara and Pellet (2000) noted that by late October/late January, the 34-month-old, juvenile *P. sachalinense* seedlings (but not the younger 10- to 22-month-old seedlings) had nearly similar CH as their 35-year-old mature parent, suggesting that attainment of physiological maturity was not a prerequisite for full expression of CA or greater CA ability of 34-month seedlings. The temporal relationship between maximum CH and flowering is likely to vary widely among woody plant species because of the great variability in the duration of the juvenile phase, which can range from 5 to 40 years among species (Kramer and Kozlowski, 1979). For example, tree genera such as *Quercus* (oak) and *Fagus* (beech) contain species with very long juvenile phases (30+ years) and it is likely in those cases that full, adult stage CH levels are attained prior to flowering.

### MECHANISMS FOR AGE-DEPENDENT PHENOTYPES

Very few studies have examined the mechanisms for age-dependent expression of adaptive traits at the whole plant, cellular, or molecular scales. There is some evidence for an association between changes in gene expression and leaf morphology/physiology during the juvenile-to-mature phase transition in woody perennials. For example, proline-rich-protein (PRP) gene is expressed more strongly in mature petioles than juvenile ones while the gene for dihydroflavonol reductase (DFR), an enzyme needed for anthocyanin production, is only present in juvenile petioles in English ivy (Murray and Hackett, 1991; Woo et al., 1994). Similarly, in both larch and English ivy, the expression of chlorophyll *a*/*b* binding protein (cab) gene decreases with maturation (Hutchison et al., 1990).

**FIGURE 5:**
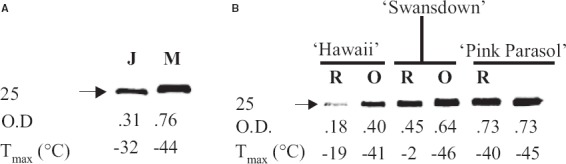
**Anti-dehydrin immunoblots of leaf protein extracts from cold-acclimated leaves (December) of different-aged wild populations of *R. maximum* seedlings (A) and clonally propagated plants of three rhododendron cultivars, *R.* “Hawaii,” *R.* “Swansdown,” and *R.* “Pink Parasol” (B).** Thirty and 15 μg total leaf proteins were loaded for immunoblots in **(A)** and **(B)**, respectively. J, juvenile seedlings (approximately 2- to 3-year-old); M, mature plants (approximately 30-year-old); R, ramet (approximately 4-year-old plants clonally propagated from the 30- to 40-year-old ortets); O, ortet; OD, optical densities (quantitative parameter for the band density); *T*_max_, quantitative measure of leaf-freezing-tolerance. Arrow points to a band of dehydrin protein of 25 kDa (this figure is adapted from Lim et al., 1999).

In an earlier report, levels of 25 kDa dehydrin (DHN) were higher (up to a 2.2-fold increase) in mature *Rhododendron* cultivars (ortets) than in 2- to 3-year-old clones (ramets) propagated as rooted cuttings from the mature plants (Lim et al., 1999; Figure 5). Similarly, 25 kDa DHN levels were 2.4-fold higher in leaves from mature wild plants of *R. maximum* than in juvenile seedlings growing nearby (Figure 5). This provides evidence of changes in gene expression associated with age-dependent CH phenotypes, because the younger clones and juvenile seedlings were also less cold-hardy than mature plants. DHN proteins, hydrophilic members of group 2 LEA family (Close, 1996), have been widely implicated to have a role in CA process and conferring protection against freeze-desiccation (Kosová et al., 2007). Furthermore, prior research established a significant correlation between the abundance of a 25-kDa DHN and CA ability of several *Rhododendron* species (Marian et al., 2004) and populations (Lim et al., 1999). At present, however, a causal relationship between plant age and 25 kDa DHN accumulation has not been established.

It has also been suggested that the relative concentrations (ratios) of phytohormones-abscisic acid (ABA), gibberellic acid (GA), auxins, and cytokinins-play a major role in regulating juvenile-to-mature phase transition (Haffner et al., 1991). The role of ABA has been well established as one of the key molecules in the signal cascade for CA in both herbaceous and woody species (Gusta et al., 2005) while other research suggests that GA is a negative regulator of cold-tolerance (Pieniazek and Holubowicz, 1973; Shan et al., 2007). Investigation into the biochemical or molecular changes vis-à-vis age-dependent changes in CH was not the focus of the present study, however, this area is ripe for in-depth research, including the study of shifts in hormonal balance and expressions of cold-regulated (cor) genes that have particularly been implicated in cold-stress tolerance.

While exploring putative mechanisms for age-dependent changes in CH (or other adaptive traits) it should be recognized that the overall size of a plant also increases with age, and, therefore, it may be difficult to separate age responses from those potentially associated with biomass accumulation. Because our estimates of CH are based on leaf freezing assays, age-dependent leaf traits could affect the results. For example, earlier work with *Rhododendron* leaves demonstrated that both leaf and cuticle thickness increased during CA and that a cold-hardy species, *R. catawbiense*, had a thicker cuticle than a less hardy species, *R. ponticum* (Wang et al., 2008). Huner et al. (1981) and Stefanowska et al. (1999) have also noted an association between CA and increased leaf-thickness, presumably due to cell wall thickness.

Our study used current year *Rhododendron* leaves to estimate CH on plants of different age and biomass. Increased leaf CH in older plants may be due in part to translocation of metabolites involved in secondary cell wall formation and lignin formation, originating from older wood cells in the stems and roots. Transcriptome comparisons of juvenile versus mature wood in *Pinus* (Li et al., 2011) has identified greater expression of genes related to cellulose synthesis and lignification in mature wood, which would make the component carbohydrates and phenols more readily available to new tissue in older plants. Alternatively, the increase in CH observed in new leaves on older plants may result from a high source:sink ratio that makes more nutrients available for stress responses. Both the biomass and metabolite explanations for age-dependent CH are intriguing but as yet untested hypotheses.

Long-lived woody plants appear to have the chemistry and adaptability to survive many types of biotic and abiotic stresses. However, despite their economic and ecological importance, particularly at maturity and beyond, there are few chronologies of stress tolerance or studies of how (or whether) these tolerances are maintained over long periods of time. It is not known, for instance, whether the observed decline in photosynthesis and stomatal conductance with increased tree age (Bond, 2000) is associated with a decline in CH or other types of stress tolerance. A better understanding of age-related CA and CH in woody plants would be of practical significance to plant breeders, tree crop producers, and foresters, and could assist ecologists in assessing the adaptability of long-lived plant communities to climate change.

### Conflict of Interest Statement

The authors declare that the research was conducted in the absence of any commercial or financial relationships that could be construed as a potential conflict of interest.

## ACKNOWLEDGMENTS

This article is dedicated to the memory of Chon-Chong (Kenny) Lim, with deep appreciation and admiration of his efforts, skills, and delightful character. We sincerely thank Mohd. Salleh Daim and Hatice S. Gulen for their technical assistance on evaluating the *Rhododendron* CH.
